# Experiences of Registered Nurses involved in regulatory disciplinary processes: Scoping review protocol

**DOI:** 10.1371/journal.pone.0360935

**Published:** 2026-10-30

**Authors:** Peggy Rauman, Robin Devey-Burry, Ahmad Deeb, Jordan Pike

**Affiliations:** 1 JBI Memorial University Nursing for Evidence-Based Healthcare: A JBI Affiliated Group, Memorial University of Newfoundland Faculty of Nursing, St. John’s, Newfoundland and Labrador, Canada; 2 Health Sciences Library, Memorial University of Newfoundland, St. John’s, Newfoundland and Labrador, Canada; King Khalid University, SAUDI ARABIA

## Abstract

**Objective:**

The objective of this scoping review is to explore available literature on the experiences of Registered Nurses (RNs), Nurse Practitioners (NPs), and Registered Psychiatric Nurses (RPNs), who have been through the disciplinary process by the regulatory body.

**Introduction:**

Nursing is a regulated profession. Professions are regulated when there is significant risk of harm to the public if the practice is performed incorrectly. Empowered by legislation, regulatory bodies have a mandate to protect the public. This is accomplished by setting and enforcing high standards of practice and intervening when a nurse’s conduct is deemed unacceptable. Existing research on disciplinary action against RNs has primarily focused on regulatory processes, predictors of misconduct, characteristics of disciplined nurses, and case outcomes across jurisdictions. However, there is a significant gap in the literature regarding the experiences of RNs have been through disciplinary proceedings. A deeper understanding of these experiences is essential to inform supportive measures for nurses during the disciplinary process and to guide improvements in regulatory policy and procedures.

**Inclusion criteria:**

This review will consider literature that includes the experiences of nurses who have gone through any component of the disciplinary process by the regulatory body. Gray literature, non-empirical, and social media (e.g., blogs) searches will be included as these sources may provide genuine and unsolicited insights into the lived experiences of nurses. Research related to the mental health of nurses, specific to disciplinary action, will be included. RNs who are regulated by entities other than a nursing regulatory body will be excluded. Research focused only on nursing disciplinary policy, procedure, outcomes or document review will be excluded, as the focus is on the lived experiences of RNs.

**Methods:**

The JBI methodology for scoping reviews will guide the review process. A 3-step search strategy will be followed to identify published and unpublished literature in English or French with no time limit placed on the search. Data extracted from eligible literature will align with the review protocol, objectives, and research question. The results will be reported in tabular and/or diagrammatic format, accompanied by a narrative summary. **OSF Review registration number:** 10.17605/OSF.IO/N3SCZ.

## Introduction

Nursing is a regulated profession. Professions are regulated when there is significant risk of harm to the public if the practice is performed incorrectly. Nursing is not the only regulated profession. Other healthcare providers who perform complex or high-risk procedures are also regulated to protect public safety. These professionals, like nurses, are also held to consistent and high standards of practice, particularly when incompetence, lack of preparation, or impairment could lead to harm [1]. Nursing is also a *self-regulated* profession. This means that rather than being directly overseen by the government, nurses are accountable to a regulatory body created by the profession itself [2]. Self-regulation is based on the idea that only members of the profession can possess the specialized knowledge, skills, and judgment required to assess the conduct of their peers, set the standards for practice, and know what is needed to maintain competence [3]. In this context, nurses are the “self” in self-regulation, meaning individual nurses are responsible for maintaining their professional knowledge, applying their skills competently, and upholding ethical and professional conduct [4].

In Canada, the regulation of nursing is governed by provincial and territorial legislation [5]. Government enacted legislation provides the legal framework for professional governance, grants nurses’ the authority to practice, and establishes regulatory bodies to oversee the profession [5]. Legislation may be specific to nursing, such as a Registered Nurses Act or part of broader umbrella legislation that includes multiple health professions, such as a Health Professions Act [2]. For example, the *Registered Nurses Act (2008)* is the legislation that governs the practice of nursing in Newfoundland and Labrador (NL). This act outlines the structures and processes for regulation, including governance frameworks, licensure requirements, continuing competency programs, education program approval, and the development and enforcement of standards of practice and codes of ethics/conduct [6]. It also establishes the procedures for addressing complaints and professional misconduct [5,7]. The provincial regulatory body is accountable to carry out the responsibilities set out in legislation by creating governance policies, regulations, regulatory processes, and organizational by-laws [5]. These regulatory documents define the requirements for nursing education program approval, entry-level competencies, standards of practice, codes of ethics/conduct, and ongoing competency requirements. Maintaining a register of licensed nurses, investigating complaints, and administering disciplinary processes maintain the public’s confidence in the profession by assurance that RNs provide safe, competent, and ethical care to the public.

Nursing regulatory bodies (RBs) have a mandate to protect the public by promoting and enforcing high standards of practice for all registered nurses and by intervening when a nurse’s conduct is deemed unacceptable [8]. The disciplinary process begins in one of two ways, either a complaint against a nurse is submitted in writing to the RB, or the RB initiates the process [9]. Complaints submitted to the nursing RB may come from individuals who are not registered nurses (e.g., members of the public), colleagues from other disciplines, or from the employer (e.g., managers, or the professional practice department) (RN Act, 2008). Upon receipt of a complaint, the RB reviews the complaint in detail to determine whether the nurse’s actions may have breached legislation, standards of practice, or the code of ethics/conduct, or posed a risk to patient safety [7]. If there is sufficient evidence to support the complaint, it must be investigated (RN Act, 2008). The RB have investigators who are authorized to collect relevant information to allow the RB to assess the evidence and make an informed decision [10]. In a systematic review of complaints against nurses, Papinaho et al. (2019) noted several categories of complaints, including violations of patient integrity inclusive of breaches of privacy and confidentiality, as well as verbal or psychological abuse. Professional misconduct complaints ranged from physical abuse, sexual misconduct, harassment, and boundary violations to theft. Nursing process complaints involved medication errors, documentation problems, failure to recognize patient signs and symptoms, misinterpretation of assessment findings, and administering care without authorization. Nursing incompetence complaints included unlicensed practice, noncompliance with regulations, and the delivery of substandard care. Other categories of complaints involved risky behaviour such as substance abuse leading to impairment, violence toward colleagues, and other forms of unprofessional behavior [4]. Impairment has been described as a decline in functioning that may interfere with a nurse’s ability to practice safely and meet professional standards. While substance abuse is commonly associated with impairment, medical conditions and other health-related issues may also contribute to impaired practice [1]. Complaint categories differ with variations in legislative and regulatory frameworks.

Disciplinary processes can result in a range of outcomes including reprimands, probation on a nurse’s licence, or limitations on practice, to suspension [4]. In some cases, an alternative dispute resolution process may be used to determine if a quality assurance (QA) approach is appropriate rather than proceeding directly to a formal disciplinary hearing. QA actions may include mandatory education, practice audits, meetings with a practice advisor, workplace supervision, or the completion of a written assignment or report [8]. More serious concerns may require a formal disciplinary hearing. A hearing typically includes a panel composed of professional peers, and a public representative who reviews evidence and witness statements to determine if the registrant engaged in conduct deserving of sanction, and if so, the appropriate discipline [11]. Sanctions resulting from a hearing may include a formal reprimand, license suspension, license revocation, or any combination of these penalties [4].

While disciplinary action is intended to protect the public, it can also lead to stigma for nurses, particularly the stigma of being blamed or labeled as unsafe [1,10]. For nurses with substance use disorders, traditional disciplinary approaches may create a barrier to seeking treatment, potentially allowing unsafe practice to continue without appropriate intervention [1]. The disciplinary process has also been criticized for lengthy investigations and legal proceedings, which may prolong risk to the public and create uncertainty for nurses under review [1]. Very little is known about, for example, how the duration of disciplinary investigations affects nurses who continue working under interim measures. When final sanctions are imposed after months or even years, the impact on a nurse’s career and well-being may be severe and long-lasting [10].

Research on disciplinary action against RNs has primarily focused on the disciplinary processes across jurisdictions, predictors and reasons for disciplinary action, characteristics of disciplined nurses, and case outcomes [1,4,8,10–12]. However, there is limited research exploring the experiences of RNs who have undergone disciplinary action.

The purpose of this scoping review is to explore available literature on the experiences of Registered Nurses (Registered Nurses (RNs) Nurse Practitioners (NPs), and Registered Psychiatric Nurses (RPNs), who have been through the disciplinary process by the regulatory body. This review will help identify gaps in the existing literature and determine whether a full systematic review is warranted. Moreover, synthesizing knowledge about RNs experiences is essential for informing the development of evidence-based recommendations to inform supportive measures for nurses during the disciplinary process and improve regulatory processes, guide policy, and support complex decision-making in professional regulation.

### Review questions

The overarching question guiding this scoping review is: What is the scope of literature on the experiences of RNs who have been through the process of disciplinary action by a nursing regulatory body?

To assist with answering this question the following sub questions will be posed:

1. What was it like to be contacted by the regulatory body with a complaint against their nursing practice?
2. What was the experience of navigating the complaints process?
3. How did the process affect the individual’s nursing practice?
4. How did the outcome affect individual’s nursing practice?

### Inclusion criteria

#### Participants.

The review will include literature on Registered Nurses (RNs, NPs, RPNs) or other national or international terminology related to RN licensure.

#### Concept.

This review aims to identify research literature and grey literature on the experiences of nurses as they progressed through the disciplinary process by the nursing regulatory body. Any part of the disciplinary process (complaint received by a member of the public, another nurse, the employer, or regulatory body; investigated and dismissed, resolved with an alterative dispute resolution, or hearing) will be included. As well, all outcomes (guilty, not guilty, quality assurance, or dismiss with reasons) will be included. RNs who are regulated by an entity other than a nursing regulatory body will be excluded.

#### Context.

This review will include all relevant qualitative research articles or unpublished review articles in English and French as these are the official languages of Canada. The context for this review will include countries with similar health care systems to Canada, and similar regulatory processes, such as the use of the National Council License Exam Registered Nurses (NCLEX - RN) to determine qualification for registration as a Registered Nurse. Articles related to the mental health of nurses specifically related to their experiences with disciplinary action will be included. Research solely focused on nursing disciplinary policies, procedures, outcomes, regulatory reports or document reviews will be excluded as the focus of this review is on the lived experiences of RNs.

#### Types of sources.

This scoping review will consider qualitative studies that focus on qualitative data including, but not limited to, designs such as phenomenology, grounded theory, ethnography, qualitative description, action research and feminist research. Mixed method designs and systematic reviews that meet the inclusion criteria will also be considered if they are relevant to the review’s questions. Gray literature, non-empirical, and social media (e.g., blogs) searches will be included as these sources may provide genuine and unsolicited insights into the lived experiences of RNs. Text, opinion papers, and unpublished dissertations/theses will also be included.

## Methods

The proposed scoping review will be conducted in accordance with the JBI methodology for scoping reviews [13] and reported in accordance with the Preferred Reporting Items for Systematic Reviews and Meta-Analysis extension for scoping reviews (PRISMA-ScR). The protocol is registered with Open Science Framework (10.17605/OSF.IO/N3SCZ).

### Search strategy

The search strategy was developed by a librarian (JP), in consultation with other team members (PR, RDB, AD). The search strategy will aim to locate both published and unpublished studies. A three-step search strategy will be utilized in this review. First an initial limited search of CINAHL Plus (EBSCO) will be conducted to identify articles on the topic. Text words contained in the titles and abstracts and index terms of relevant articles from the initial search, were used to develop a full search strategy for CINAHL Plus (EBSCO). The full search strategy is provided in S1 Appendix – Table 1. The search strategy, including all identified keywords and index terms, will be adapted for each included database and/or source of information. Databases to be searched include CINAHL, MEDLINE (OVID), APA PsycINFO (EBSCO), JBI EBP database, Nursing and Allied Health database, and Academic Search Complete. Sources of unpublished/grey literature will include ProQuest Dissertations and Theses, Google, and relevant social media public opinion websites. Backward and forward citation tracing of included articles will be performed. If the full text of any included source of information cannot be accessed or the team has questions about the source of evidence, then the team will attempt to contact the author via author’s contact email. Attempts to contact and successful contact with authors via email and the outcome of the contact will be summarized in an appendix. Literature published in English and French will be included. There are no limits on publication dates as historical trends may be relevant.

### Source of Evidence Selection

Following the search, all identified citations will be collated and uploaded into Covidence systematic review software (Veritias Health Innovation, Melbourne, Australia) and duplicated records will be removed. To assess reviewer agreement regarding relevance of titles and abstracts, 5–10 articles will be tested prior to the full title and abstract screening. Once reviewer agreement is established, titles and abstracts will be screened by two or more reviewers for assessment against the inclusion criteria for the review. Potentially relevant sources will be retrieved in full. The full text review of selected citations will be assessed in detail against the inclusion criteria by two or more reviewers. Reasons for exclusion of sources of evidence at full text that do not meet inclusion criteria will be recorded and reported in the scoping review and the source of evidence will be removed. Any disagreements that arise between reviewers at any stage of the selection process will be resolved through discussion and agreement, or by involving additional reviewer/s. Full text review is anticipated to be completed within one month. The results of the search and the inclusion process will be reported in full in the final scoping review using a PRISMA flow diagram.

### Data extraction

Data will be extracted from sources of evidence included in the scoping review by two or more independent reviewers using a data extraction tool developed by the reviewers. A draft data extraction form is provided in S2 Appendix – Table 2. To assess the accuracy of the data extraction tool and to assess agreement between reviewers, a pilot test of 3–5 sources of evidence will be conducted prior to full data extraction. The draft data extraction tool will be modified and revised as necessary during the process of extracting data from each included evidence source. Modifications will be detailed in the scoping review. Any disagreements or bias that arise between the reviewers will be resolved through discussion, or with an additional reviewer/s.

The data extracted will include specific details about the participants, concept, context, study methods and key findings relevant to the review question/s.

This scoping review protocol will commence on December 1, 2026. Following completion of the search strategy, title and abstract screening by two independent reviewers is anticipated to be completed within 4–6 weeks, followed by full-text screening over the subsequent 2–3 weeks. Data extraction (charting) and verification are expected to be completed within 6–8 weeks, with data synthesis and preparation of the final report occurring thereafter. It is anticipated that the findings of the scoping review will be completed and ready for dissemination by March 2027

### Data analysis and presentation

The PRISMA-ScR guidelines will be followed in the presentation of results. Data extracted will be presented in a table and will include details from the data extraction tool such as country of origin, study objectives, nature of complaints against nurses, emotions associated with complaints, complaints processes, duration of process, coping strategies used by nurses, and recommendations for nursing regulators. A narrative summary describing how the results relate to the review questions and objective of the scoping review will also be presented.

## Supporting information

S1 Checklist Checklist_PRISMA-P 2015_Rauman. (DOCX)

S1 Appendix Search strategy and number of records retrieved. **CINAHL Plus (EBSCO), February 6, 2026.** The search strategy comprised three main concepts: (1) professional discipline and disciplinary action, (2) nursing organizations and professional regulation, and (3) nurses and nursing practice. Search terms within and across concepts were combined using Boolean operators (AND/OR), with truncation used to capture variations of search terms. The final search set (S13) combined the professional discipline/regulation concepts with the nursing population/practice concept and was limited to publications in English or French. The number of records retrieved at each stage of the search is reported in the Results column. Database-specific search field codes are retained as displayed in the original search strategy.
(TIF)

S2 Appendix Data extraction tool for the scoping review. The tool is to systematically extract information from included evidence sources regarding nurses’ experiences with regulatory discipline. Data included source and participant characteristics, nature of the complaint, and evidence related to experiences of receiving a complaint, being investigated, working during an investigation, and experiencing the outcome of the disciplinary process. Data also extracted on emotions, mental health, coping strategies, short- and long-term effects on nursing practice, and implications. RN, registered nurse; NP, nurse practitioner; RPN, registered psychiatric nurse.
(TIF)
