# Supplementary material for: Experiences of Registered Nurses involved in regulatory disciplinary processes: Scoping review protocol
Source: PLoS One. 2026 Oct 30;21(10):e0360935. doi: 10.1371/journal.pone.0360935 (PMC13722312; doi:10.1371/journal.pone.0360935)
Supplement: Supplementary file 2 [file pone.0360935.s004.docx]

**Faculty of Nursing**

300 Prince Philip Drive

St. John’s, NL Canada A1B 3V6

Tel: 709 864 2719 Fax: 709 864 8814

www.mun.ca/nursing

September 29, 2026

Dear PLOS One,

Please accept this letter of response to the editorial revision request dated September 22, 2026, to our manuscript entitled Experiences of Registered Nurses Involved in Regulatory Disciplinary Processes: Scoping Review Protocol for publication as a study protocol.

Item 1 – Figures were removed from the manuscript file, leaving only the individual TIFF/EPS image files. This is represented in the tracked changes version of the manuscript file and the clean version of the manuscript file dated for September 29, 2026.

Item 2 – **Legends were included for figures 1 and 2 in a separate document. It was uploaded as other and titled Legends dated for September 29, 2026.**

Thank you for the opportunity to provide the revisions.

Sincerely,

Peggy Rauman, RN, PhD

Memorial University of Newfoundland

Faculty of Nursing /prauman@mun.ca

Received Tuesday August 11^th^, 2026

PLOS ONE Decision: Revision required [PONE-D-26-29284] - [EMID:7389dcbbba3d7e2f

**ACADEMIC EDITOR:**

**Subject: Feedback on Scoping Review Protocol-PONE-D-26-29284 (Minor Revisions Needed Before decision)**

Dear Dr. Rauman,

Thank you for sharing the protocol manuscript. Overall, it is well-structured and methodologically sound, following JBI scoping review methodology and PRISMA-ScR reporting standards appropriately. Before submission, please address the following two issues:

**1. Title inconsistency between cover letter and manuscript**
The cover letter states the title as 'Nurses' Experiences of Disciplinary Action by Nursing Regulatory Bodies: A Scoping Review Protocol,' while the manuscript title page reads 'Experiences of Registered Nurses Involved in Regulatory Disciplinary Processes: Scoping Review Protocol.' These must match exactly, as editorial offices routinely check for this. Please confirm which title is final and update the other document accordingly.

**2. Duplicated/garbled phrasing in the Objective statement**
Both the submission-system Abstract and the in-manuscript Abstract (line 35–38) contain the following text:

'...experiences of Registered Nurses (Registered Nurses (RNs) Nurse Practitioners (NPs), and Registered Psychiatric Nurses (RPNs)...'

This appears to be a copy-paste artifact  'Registered Nurses' is duplicated, there's a stray open parenthesis, and a comma is missing after '(RNs).' The corrected version should read:

'...experiences of Registered Nurses (RNs), Nurse Practitioners (NPs), and Registered Psychiatric Nurses (RPNs)...'

Please correct this in both locations.

All other required elements. Financial Disclosure, Competing Interests, Ethics Statement, Data Availability Statement, OSF prospective registration, and the PRISMA-P checklist, meet PLOS ONE's requirements for a Study Protocol resubmission.

==============================
