# Supplementary material for: Experiences of Registered Nurses involved in regulatory disciplinary processes: Scoping review protocol
Source: PLoS One. 2026 Oct 30;21(10):e0360935. doi: 10.1371/journal.pone.0360935 (PMC13722312; doi:10.1371/journal.pone.0360935)
Supplement: Peer review history

# Editor report: Decision Letter 0

**DOI:** 10.1371/journal.pone.0360935.r001

**Authors:** Shadia Hamoud Alshahrani

11 Aug 2026

PONE-D-26-29284

Experiences of Registered Nurses Involved in Regulatory Disciplinary Processes: Scoping Review Protocol

PLOS One

Dear Dr. Rauman,

Thank you for submitting your manuscript to PLOS ONE. After careful consideration, we feel that it has merit but does not fully meet PLOS ONE’s publication criteria as it currently stands. Therefore, we invite you to submit a revised version of the manuscript that addresses the points raised during the review process.

==============================

**ACADEMIC EDITOR:**

**Subject: Feedback on Scoping Review Protocol-PONE-D-26-29284 (Minor Revisions Needed Before decision)**

Dear Dr. Rauman,

Thank you for sharing the protocol manuscript. Overall, it is well-structured and methodologically sound, following JBI scoping review methodology and PRISMA-ScR reporting standards appropriately. Before submission, please address the following two issues:

**1. Title inconsistency between cover letter and manuscript**

The cover letter states the title as *'Nurses' Experiences of Disciplinary Action by Nursing Regulatory Bodies: A Scoping Review Protocol,'*  while the manuscript title page reads *'Experiences of Registered Nurses Involved in Regulatory Disciplinary Processes: Scoping Review Protocol.'*  These must match exactly, as editorial offices routinely check for this. Please confirm which title is final and update the other document accordingly.

**2. Duplicated/garbled phrasing in the Objective statement**

Both the submission-system Abstract and the in-manuscript Abstract (line 35–38) contain the following text:

'...experiences of Registered Nurses (Registered Nurses (RNs) Nurse Practitioners (NPs), and Registered Psychiatric Nurses (RPNs)...'

This appears to be a copy-paste artifact  'Registered Nurses' is duplicated, there's a stray open parenthesis, and a comma is missing after '(RNs).' The corrected version should read:

'...experiences of Registered Nurses (RNs), Nurse Practitioners (NPs), and Registered Psychiatric Nurses (RPNs)...'

Please correct this in both locations.

All other required elements. Financial Disclosure, Competing Interests, Ethics Statement, Data Availability Statement, OSF prospective registration, and the PRISMA-P checklist, meet PLOS ONE's requirements for a Study Protocol resubmission.

==============================

Please submit your revised manuscript by Sep 09 2026 11:59PM. If you will need more time than this to complete your revisions, please reply to this message or contact the journal office at plosone@plos.org. When you're ready to submit your revision, log on to https://www.editorialmanager.com/pone/ and select the 'Submissions Needing Revision' folder to locate your manuscript file.

Please include the following items when submitting your revised manuscript:

- A letter that responds to each point raised by the academic editor and reviewer(s). You should upload this letter as a separate file labeled 'Response to Reviewers'.
- A marked-up copy of your manuscript that highlights changes made to the original version. You should upload this as a separate file labeled 'Revised Manuscript with Track Changes'.
- An unmarked version of your revised paper without tracked changes. You should upload this as a separate file labeled 'Manuscript'.

you would like to make changes to your financial disclosure, please include your updated statement in your cover letter. Guidelines for resubmitting your figure files are available below the reviewer comments at the end of this letter.

If applicable, we recommend that you deposit your laboratory protocols in protocols.io to enhance the reproducibility of your results. Protocols.io assigns your protocol its own identifier (DOI) so that it can be cited independently in the future. For instructions see: https://journals.plos.org/plosone/s/submission-guidelines#loc-laboratory-protocols. Additionally, PLOS ONE offers an option for publishing peer-reviewed Lab Protocol articles, which describe protocols hosted on protocols.io. Read more information on sharing protocols at https://plos.org/protocols?utm_medium=editorial-email&utm_source=authorletters&utm_campaign=protocols.

As the corresponding author, your ORCID iD is verified in the submission system and will appear in the published article. PLOS supports the use of ORCID, and we encourage all coauthors to register for an ORCID iD and use it as well. Please encourage your coauthors to verify their ORCID iD within the submission system before final acceptance, as unverified ORCID iDs will not appear in the published article. *Only*  the individual author can complete the verification step; PLOS staff *cannot*  verify ORCID iDs on behalf of authors.

We look forward to receiving your revised manuscript.

Kind regards,

Shadia Hamoud Alshahrani, PhD

Academic Editor

PLOS One

Journal Requirements:

When submitting your revision, we need you to address these additional requirements.

1. Please ensure that your manuscript meets PLOS ONE's style requirements, including those for file naming. The PLOS ONE style templates can be found at

https://journals.plos.org/plosone/s/file?id=wjVg/PLOSOne_formatting_sample_main_body.pdf and

https://journals.plos.org/plosone/s/file?id=ba62/PLOSOne_formatting_sample_title_authors_affiliations.pdf

2. Thank you for stating the following in the Competing Interests section:

“I have read the journal's policy and the corresponding authors of this manuscript have the following competing interests: the corresponding author worked as a nursing consultant for policy and practice for the provincial regulatory body from 2018-2023. This could be perceived as a competing interest.”

Please confirm that this does not alter your adherence to all PLOS ONE policies on sharing data and materials, by including the following statement: 'This does not alter our adherence to  PLOS ONE policies on sharing data and materials.” (as detailed online in our guide for authors http://journals.plos.org/plosone/s/competing-interests).  If there are restrictions on sharing of data and/or materials, please state these. Please note that we cannot proceed with consideration of your article until this information has been declared.

Please include your updated Competing Interests statement in your cover letter; we will change the online submission form on your behalf.

3. Please ensure that you refer to Figures 1 and 2 in your text as, if accepted, production will need this reference to link the reader to the figure.

4. Please include captions for your Supporting Information files at the end of your manuscript, and update any in-text citations to match accordingly. Please see our Supporting Information guidelines for more information: http://journals.plos.org/plosone/s/supporting-information.

5. If the reviewer comments include a recommendation to cite specific previously published works, please review and evaluate these publications to determine whether they are relevant and should be cited. There is no requirement to cite these works unless the editor has indicated otherwise.

6. Please review your reference list to ensure that it is complete and correct. If you have cited papers that have been retracted, please include the rationale for doing so in the manuscript text, or remove these references and replace them with relevant current references. Any changes to the reference list should be mentioned in the rebuttal letter that accompanies your revised manuscript. If you need to cite a retracted article, indicate the article’s retracted status in the References list and also include a citation and full reference for the retraction notice.

[Note: HTML markup is below. Please do not edit.]

[NOTE: If reviewer comments were submitted as an attachment file, they will be attached to this email and accessible via the submission site. Please log into your account, locate the manuscript record, and check for the action link "View Attachments". If this link does not appear, there are no attachment files.]

To ensure your figures meet our technical requirements, please review our figure guidelines: https://journals.plos.org/plosone/s/figures

You may also use PLOS’s free figure tool, NAAS, to help you prepare publication quality figures: https://journals.plos.org/plosone/s/figures#loc-tools-for-figure-preparation.

NAAS will assess whether your figures meet our technical requirements by comparing each figure against our figure specifications.

# Author comment: Author response to Decision Letter 1

**DOI:** 10.1371/journal.pone.0360935.r002

30 Sep 2026

Dear PLOS One,

Please accept this letter of response to the editorial revision request dated Tuesday, August 11, 2026, to our manuscript entitled Experiences of Registered Nurses Involved in Regulatory Disciplinary Processes: Scoping Review Protocol for publication as a study protocol.

Item 1 - title inconsistency between cover letter and manuscript has been corrected to reflect the confirmed and final title: Experiences of Registered Nurses Involved in Regulatory Disciplinary Processes: Scoping Review Protocol.

Item 2 - Duplicated/garbled phrasing in the Objective statement (line 35 -38) has been correct to remove the duplicate registered nurse phrase, and a comma was added after (RN). Initially I wanted to convey that there are three regulated registered nursing designations including registered nurse, nurse practitioner and registered psychiatric nurse and that was the reason for the extra bracket and duplication of the words registered nurse. I forgot to put the comma in after (RN). However, once I corrected the sentence as per your suggestion, it was clearer and more concise. Thank you.

As per your instructions, a tracked changes and clean version of both the manuscript and the cover letter have been uploaded for your review and accompany this letter.

Attachment Submitted filename: Response Letter Sep 29.docx

# Editor report: Decision Letter 1

**DOI:** 10.1371/journal.pone.0360935.r003

**Authors:** Shadia Hamoud Alshahrani

5 Oct 2026

Experiences of Registered Nurses Involved in Regulatory Disciplinary Processes: Scoping Review Protocol

PONE-D-26-29284R1

Dear Dr. Peggy Rauman

<table border="0" cellpadding="0" cellspacing="0" class="datatable3"><tbody><tr><td style="width:30%"> </td><td style="text-align:left;"> </td></tr></tbody></table>

We’re pleased to inform you that your manuscript has been judged scientifically suitable for publication and will be formally accepted for publication once it meets all outstanding technical requirements.

Within one week, you’ll receive an e-mail detailing the required amendments. When these have been addressed, you’ll receive a formal acceptance letter and your manuscript will be scheduled for publication.

An invoice will be generated when your article is formally accepted. Please note, if your institution has a publishing partnership with PLOS and your article meets the relevant criteria, all or part of your publication costs will be covered. Please make sure your user information is up-to-date by logging into Editorial Manager at Editorial Manager® and clicking the ‘Update My Information' link at the top of the page. For questions related to billing, please contact billing support.

If your institution or institutions have a press office, please notify them about your upcoming paper to help maximize its impact. If they’ll be preparing press materials, please inform our press team as soon as possible -- no later than 48 hours after receiving the formal acceptance. Your manuscript will remain under strict press embargo until 2 pm Eastern Time on the date of publication. For more information, please contact onepress@plos.org.

Kind regards,

Shadia Hamoud Alshahrani, PhD

Academic Editor

PLOS One

# Editor report: Acceptance letter

**DOI:** 10.1371/journal.pone.0360935.r004

**Authors:** Shadia Hamoud Alshahrani

PONE-D-26-29284R1

PLOS One

Dear Dr. Rauman,

I'm pleased to inform you that your manuscript has been deemed suitable for publication in PLOS One. Congratulations! Your manuscript is now being handed over to our production team.

At this stage, our production department will prepare your paper for publication. This includes ensuring the following:

* All references, tables, and figures are properly cited

* All relevant supporting information is included in the manuscript submission,

* There are no issues that prevent the paper from being properly typeset

You will receive further instructions from the production team, including instructions on how to review your proof when it is ready. Please keep in mind that we are working through a large volume of accepted articles, so please give us a few days to review your paper and let you know the next and final steps.

Lastly, if your institution or institutions have a press office, please let them know about your upcoming paper now to help maximize its impact. If they'll be preparing press materials, please inform our press team within the next 48 hours. Your manuscript will remain under strict press embargo until 2 pm Eastern Time on the date of publication. For more information, please contact onepress@plos.org.

You will receive an invoice from PLOS for your publication fee after your manuscript has reached the completed accept phase. If you receive an email requesting payment before acceptance or for any other service, this may be a phishing scheme. Learn how to identify phishing emails and protect your accounts at https://explore.plos.org/phishing.

If we can help with anything else, please email us at customercare@plos.org.

Thank you for submitting your work to PLOS One and supporting open access.

Kind regards,

PLOS One Editorial Office Staff

on behalf of

Dr. Shadia Hamoud Alshahrani

Academic Editor

PLOS One
